# Death from Tubal Pregnancy

**Published:** 1866-05

**Authors:** A. Fisher

**Affiliations:** Chicago, Ill.


ARTICLE XVIII.

### DEATH FROM TUBAL PREGNANCY.

By A. FISHER, M.D., Chicago, Ill.

Read to the Chicago Medical Society, March 23d, 1866

*May 31st, 1860.*—I was called to visit Mrs. B., aged 31, sanguine nervous temperament; has given birth to four children; has not menstruated for three months, consequently supposed to be between two and three months advanced in pregnancy. She complained of neuralgic pains in the back, which subsided in a few days under ordinary treatment.

*June 7th,* one week after, between 12 and 1 o'clock P.M., I was called in haste to see her again. She grew worse so fast, after they sent for me, that Dr. GROESBECK was called in to see her before I arrived, which was about 1 P.M., when I obtained the following history of the attack:—She was quite well the day previous, and on that day, until about 11 o'clock, when she was taken with severe pain in the bowels, with a desire to go to stool. Soon after, while in the water closet on the vessel, the pain increased and she was quite faint, so that she had to call for assistance to get in bed. Dr. G. said, when he first arrived, about half-an-hour previously, that she was very faint and much prostrated. He had given morphine and stimulants, and had applied cataplasms over the stomach and bowels. At that time, her pulse was 130, very weak and feeble; countenance pale and exsanguineous; extremities cool, and wet with perspiration; having a great and almost uncontrollable desire to get up and evacuate the bladder, which we dare not permit for fear of fatal syncope. The abdomen being somewhat distended, with no tympanitis, and the desire to urinate so great, that we introduced the catheter, to make it certain that the restlessness was not in a measure caused by distention of the bladder; but not a spoonful of urine was obtained. Tinc. opium, carbonate of ammonia, tinc. cantharides, brandy, &c., were freely administered, with very little effect. About 4 o'clock

P.M., Professor BYFORD saw her in consultation. He advised the continuation of stimulants, cataplasms, &c., as the only possible hope, but did not pretend to diagnose the case. All we could say, was that the symptoms were such as usually occur in internal hemorrhage, or rupture of some internal organ. She continued to sink, having the same desire to evacuate the bowels and bladder, with occasional cramps of the extremities, until about 7 o'clock P.M., when death ended her sufferings.

*Post mortem* examination was made the next day, by Professor BYFORD and myself. On laying open the abdomen, we found, in the peritoneal cavity, between four and five quarts of blood, which was about half coagulated. After removing it and sponging out the cavity, we found the left fallopian tube much enlarged and ruptured, containing a male fœtus, about one and a-half inches in length. The only artery ruptured was not larger than an ordinary-sized knitting-needle. The uterus was slightly enlarged, but was not lined with the membrana decidua, as it sometimes is in tubal pregnancies. Every other organ in the abdominal cavity appeared perfectly normal.

This case I ought to have reported at the time of its occurrence, and intended to do so, but neglected it from time to time until it was almost forgotten. A few days since, I heard of a similar case, which reminded me of it, and I determined to report it at once.

I believe it is the duty of physicians, in such cases, or any case, of sudden death, where the cause is not perfectly evident, to propose and insist upon a *post mortem* examination, and report the case, for numerous reasons:—First, to exonerate the physician and attendants from all blame. Secondly, to quiet the minds of all with regard to the cause of death, showing by an examination, in many cases, that no remedies, however judiciously prescribed, could have saved the patient. Finally, by recording the symptoms and treatment of the case, and the appearances after death, we have additional facts to assist us in diagnosing similar cases.

Professor HODGE says, in his *System of Obstetrics*, that there seems to be limit to the development of the Fallopian tube, so that rupture usually occurs in tubal pregnancies before the fifth month, and that the patient generally perishes from hemorrhage or inflammation. He further says, that M. HECKER has shown that the fatality in tubal pregnancies is about 63 to 1, or about 98½ per cent, while in primitive abdominal pregnancies, the fatality is 56 to 76, or nearly 42½ per cent.

Undoubtedly many women perish from hemorrhage or inflammation, occasioned by extra uterine pregnancies, without any suspicion of the cause, and if *post mortem* examinations were made, in every case, it would unquestionably reveal the fact that a much greater number of deaths occur from that cause than are shown by statistics, consequently it is impo,sible to ascertain the percentage of deaths from extra uterine pregnancy.

